# Successful Termination of Conjoined Twins in the Second Trimester: A Case Report

**DOI:** 10.7759/cureus.85309

**Published:** 2025-06-03

**Authors:** Ibrahim Kale

**Affiliations:** 1 Obstetrics and Gynecology, Umraniye Training and Research Hospital, Istanbul, TUR

**Keywords:** case report, conjoined twins, medical abortion, pregnancy, thoracic-omphalopagus conjoined twins

## Abstract

We report a case of 19-week gestation thoracic-omphalopagus conjoined twins successfully terminated via surgical evacuation following medical abortion. A 32-year-old woman (G2P1) was referred to Umraniye Training and Research Hospital for pregnancy termination after the diagnosis of conjoined twins. Following the family's decision, cervical ripening was achieved with misoprostol. After the medical abortion, a revision curettage was performed to remove residual placental tissue and fetal membranes. The patient was discharged the following day without complications. Conjoined twins are rare and generally associated with a poor prognosis. Although early terminations are typically uncomplicated, advancing gestational age increases maternal morbidity and may require cesarean delivery for termination.

## Introduction

Conjoined twins are defined as twins that are physically fused both in utero and at birth. This occurs when a monozygotic twin pregnancy divides more than 13 days post-fertilization. Conjoined twins are extremely rare, with an incidence ranging from one in 50,000 to 200,000 live births [1]. Conjoined twinning occurs more frequently in females, with a female-to-male ratio 3:1 [2]. There are no reports in the literature of familial aggregation of conjoined twins, nor any documented associations with other unrelated congenital anomalies [3].

The most common types of conjoined twins include thoraco-omphalopagus (joined at the thorax and abdomen) (28%), thoracopagus (joined at the thorax) (18.5%), omphalopagus (joined at the abdomen) (10%), heteropagus (parasitic) (10%), and craniopagus (joined at the cranium) (6%). Less common types include pyopagus (joined at the sacrum and perineum), rachipagus (joined at the vertebral column), ischiopagus (joined at the lower abdomen and pelvis), and cephalopagus (joined from the head to the umbilicus) [4]. Conjoined twins not only face the challenge of shared organs but are also often associated with significant congenital anomalies [5].

Although a detailed ultrasound examination in the late first or early second trimester is generally sufficient to diagnose conjoined twins, magnetic resonance imaging (MRI) may be necessary in cases of advanced gestational age, maternal obesity, or oligohydramnios, where sonographic imaging may not provide adequate anatomical detail. MRI is also valuable when the pregnancy is expected to continue and postnatal surgical separation is being considered, due to its superior soft tissue contrast and anatomical resolution [6,7].

The prognosis of conjoined twins largely depends on the location of fusion and the extent of shared vital organs. In some cases, conjoined twins are associated with congenital anomalies that are incompatible with life [8]. Even in the absence of such anomalies, these cases are frequently characterized by high perinatal morbidity and mortality rates, often leading families to opt for pregnancy termination [9]. However, due to the extreme rarity of conjoined twins, there is no consensus on the most appropriate method for pregnancy termination in advanced gestational weeks.

Here, we present a case of 19-week thoraco-omphalopagus conjoined twins successfully terminated via revision curettage following a medical abortion.

## Case presentation

A 32-year-old pregnant woman with a gravida 2, parity one obstetric history was admitted to the Obstetrics and Gynecology Department of Umraniye Training and Research Hospital, Istanbul, for pregnancy termination due to conjoined twins. According to the patient's anamnesis, she was 19 weeks pregnant based on her last menstrual period. The patient had no systemic diseases, did not smoke, did not consume alcohol, and her previous pregnancy, which resulted in a cesarean delivery three years ago, was uneventful. She did not attend antenatal check-ups during the first trimester of this pregnancy, and it was only discovered that she was carrying conjoined twins during an ultrasound examination conducted one week before her referral to our clinic.

A detailed obstetric ultrasound revealed that both fetuses' biparietal diameters and femur lengths were consistent with 19 weeks of gestation, and they were conjoined at the thorax and upper abdomen. The conjoined twins were male. Both fetuses had separate hearts and pericardia, but their livers were fused. Fetus 1 was found to have an atrioventricular septal defect, while fetus 2 had bilateral pelvic kidneys, a mega bladder, and bilateral pelviectasis. Fetal karyotyping was offered, but the family declined.

**Figure 1:**
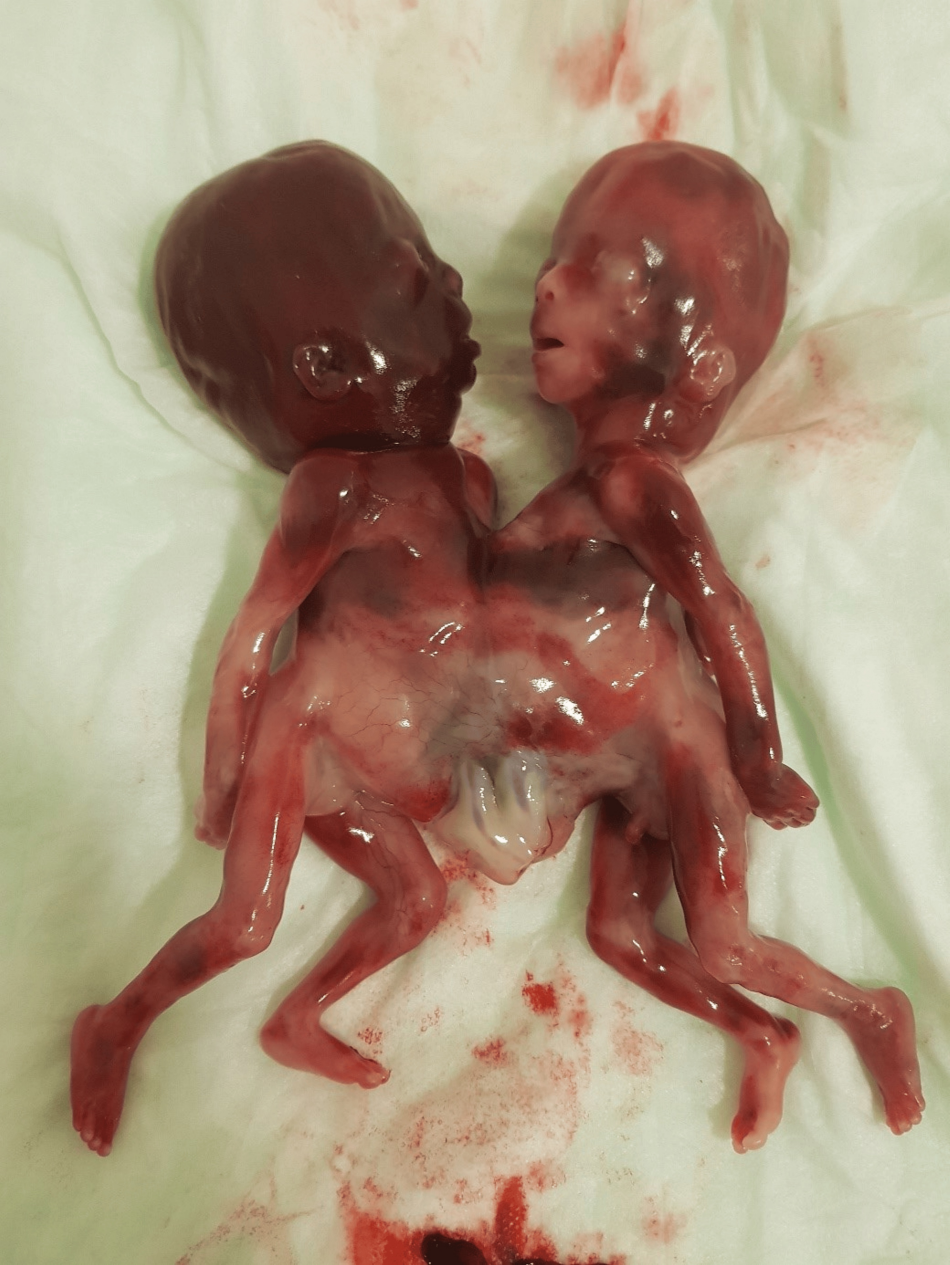
Thoraco-omfalopagus conjoined twins at 19 weeks of gestation.

A multidisciplinary team of obstetricians and fetal medicine specialists provided counseling. After discussing the prognosis and available treatment options, the family opted to terminate the pregnancy. Following informed consent, cervical preparation was carried out with one sublingual and one vaginal dose of misoprostol every four hours. Medical abortion occurred 12 hours after the first dose of misoprostol (Figure 1). Revision curettage was performed using a Carmen cannula under general anesthesia to remove the remaining placental tissue and fetal membranes. The twins were handed over to the family, as they declined pathological or genetic examination. The patient was discharged the day after the uneventful procedure.

## Discussion

Conjoined twins are extremely rare; however, with today's advanced diagnostic techniques, they can be diagnosed as early as the 12th week of pregnancy [7]. Although the diagnosis is generally made in the second trimester, reports from developing countries suggest that the diagnosis of conjoined twins may be delayed until the third trimester or even until delivery [10,11]. Early diagnosis allows valuable time for prenatal counseling, planning for delivery, and postnatal management. Detailed prenatal anatomical screening helps define the extent of organ sharing and provides valuable information for counseling [12]. Prenatal counseling is particularly crucial given the generally poor prognosis of conjoined twins. It is estimated that approximately 40% of conjoined twins are stillborn, and an additional 35% die within the first 24 hours after birth. The survival rate is higher among those who survive long enough to undergo surgery, ranging from 64% to 80% [13].

In conjoined twins, key considerations when counseling the family include the fusion site, associated malformations, the extent and type of cardiac fusion, the feasibility of surgical separation, and the expected survival rates. In particular, thoracopagus cases, which often involve pericardial fusion or significant cardiac sharing, along with accompanying cardiac anomalies, are associated with a poor prognosis [14].

After the family is thoroughly informed about the prognosis of conjoined twins, cesarean delivery is recommended in cases where a trial of life is decided. For conjoined twins in the third trimester, cesarean section is the preferred mode of delivery, regardless of the planned neonatal care approach [13-15]. In conjoined twins with a viable life expectancy, delivery should occur in a tertiary care center with a multidisciplinary team, including neonatologists, pediatric surgeons, and social services [13].

Cesarean section for conjoined twins is generally well tolerated, and most cases do not experience complications specific to the birth of conjoined twins. There are reports in the literature suggesting that vaginal delivery of conjoined twins is possible; however, most of these cases occur when prenatal diagnosis has not been made [16]. Potential complications of vaginal delivery include trauma to the birth canal, uterine rupture, and dystocia [13].

Gestational age is a critical factor in determining the method of pregnancy termination in cases of conjoined twins. While the termination of conjoined twins in early gestation is typically managed with a simple medical abortion, the risk of obstructive complications and maternal birth canal injury during delivery increases significantly in later gestational weeks, often necessitating cesarean section [14]. Safe medical termination of conjoined twins with no significant maternal morbidity has been reported up to the late second trimester [17]. A 2021 review reported that approximately 75% of terminations in conjoined twins were managed with vaginal delivery after medical induction, while cesarean section was performed in 18% of cases [9].

## Conclusions

Conjoined twins are rare, and the overall prognosis is generally poor. Terminations in the early weeks are typically uneventful; however, maternal morbidity increases with advanced gestational age, and a cesarean section may be required. For families who do not wish to pursue termination, accurate prenatal assessment and management, as well as delivery by an expert multidisciplinary team, are essential for the care of conjoined twins.
